# Prediction of habitat suitability for *Patrinia sibirica* Juss. in the Southern Urals

**DOI:** 10.1038/s41598-021-99018-0

**Published:** 2021-10-04

**Authors:** Nikolai Fedorov, Aliya Kutueva, Albert Muldashev, Oksana Mikhaylenko, Vasiliy Martynenko, Yulia Fedorova

**Affiliations:** 1 Ufa Institute of Biology - Subdivision of the Ufa Federal Research Centre of the Russian Academy of Sciences, Ufa, Russia 450054; 2 Ufa State Petroleum Technological University, Ufa, Russia 450064

**Keywords:** Biodiversity, Climate-change ecology, Conservation biology

## Abstract

The paper presents the results of predictions of the habitat persistence for rare relict of the Pleistocene floristic complex *Patrinia sibirica* (L.) Juss. in the Southern Urals under various forecasted climate change scenarios. Climate variables from CHELSA BIOCLIM, elevation data (GMTED2010) and coarse fragment content in the top level of soil were used as predictors for modeling in the MaxEnt software. The impact of climate change on *P. sibirica* habitats under the RCP4.5 and RCP8.5 scenarios calculated from an ensemble of four general circulation models has been analyzed. The modeling has shown that the changes in the habitat suitability depend on the altitude. Deterioration of the habitats could be attributed to a temperature increase in mountain forest locations, and to a precipitation of driest quarter increase in mountain forest-steppe locations. In both cases, this leads to the expansion of forest and shrub vegetation. Monitoring of the habitat persistence of *P. sibirica* and other relict species of the Pleistocene floristic complex can play a major role in predictions, as their massive decline would constitute that climatic changes exceed the ranges of their fluctuations in the Holocene.

## Introduction

**Figure 1:**
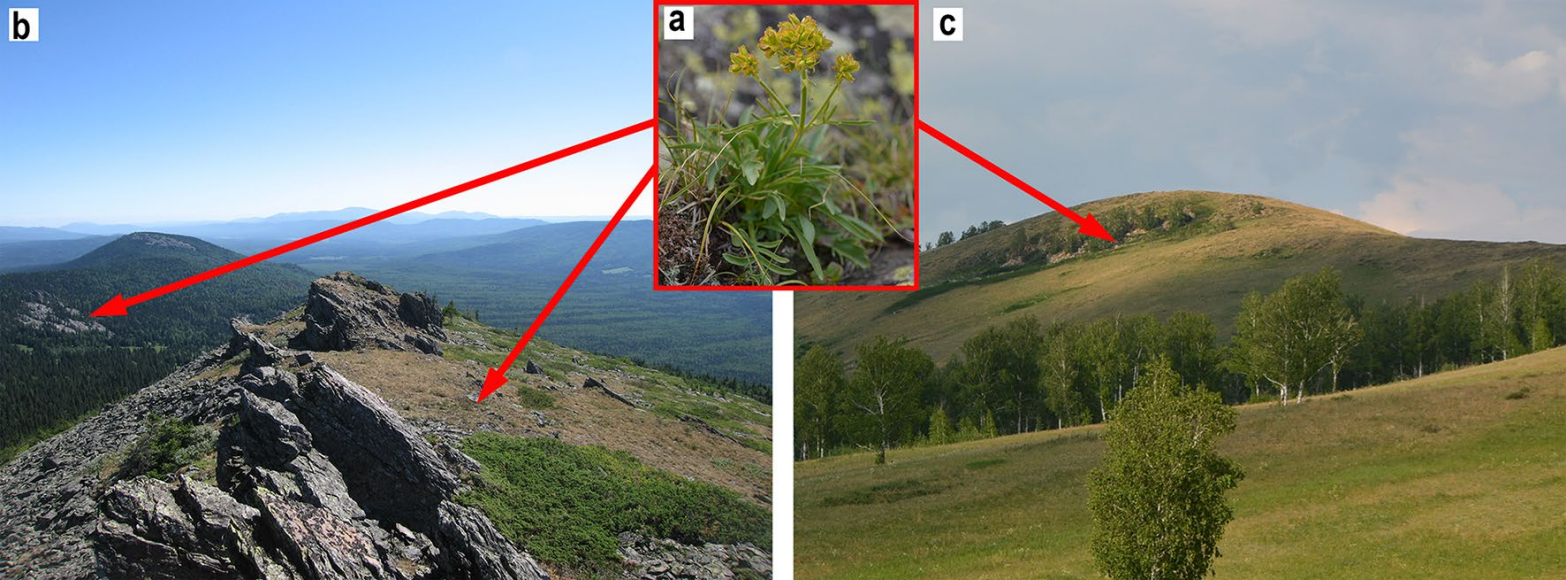
*Patrinia sibirica* (**a**) and its typical habitats in mountain forest (**b**) and mountain forest-steppe (**c**) zones.

Environmental modeling is increasingly used for environmental policy formulation and making decisions on rare species protection measures including those under climate change^1–3^. The need for modeling of climate change impact on plant habitats is particularly evident for predictions of the stability of potentially most vulnerable rare species, including relicts of the Pleistocene floristic complex in the Southern Urals (Russia). One of these species is *Patrinia sibirica* (L.) Juss. (Valerianaceae), which grows on the western border of its current range in the Southern and Middle Urals^4,5^. *P. sibirica* grows on rocky, well-insulated mountain slopes, flat ridge tops and rocky steppes^6^. The species is included in the Red Data Books of the Chelyabinsk Region^7^ and the Republic of Bashkortostan^8^. It is considered to be a Pleistocene mountain steppe relict of south Siberian origin^5^. However, *P. sibirica* is also found in the most elevated parts of the Southern Urals where the climate is quite cold and humid. Thus, it belongs to the group of species with two optimum growth locations: one is in the mountain forest-steppe areas and another one is in the subalpine region (Fig. 1). It apparently had a fairly wide distribution in the ancient boreal xerophyte biotopes of the Pleistocene floristic complex. This group of plants in the Southern Urals also includes such species as *Allium strictum* Schrad., *Bupleurum multinerve* DC., *Carex pediformis* C.A.Mey., *Scorzonera glabra* Rupr. and some others^4,5^.

It is known that stenotopic species including *P. sibirica* grow under specific edaphic conditions (edaphic specialist species) and usually have a number of stress-tolerant functional features such as resistance to extreme humidity^9,10^. Perhaps one of the adaptation mechanisms of *P. sibirica* is high rate of seasonal growth^11^. This allows the plants to adapt to the relatively short growing season in the mountains and in the steppe before the dry season starts. Their adaptation to drought could also be explained because rocky habitats often have remarkable edaphic heterogeneity, including small-scale variabilities in soil moisture^12,13^ and temperature^14^.

## Results

The resulting model of the current potential distribution area of *P. sibirica* in the Southern Urals has a mean AUC of 0.989, which corresponds to high quality of the model. The resulting model was based on six variables. Isothermality (Bio3) and temperature seasonality (Bio4) had the largest contribution to the model, accounting for 29.3% and 25.9%, respectively. Both parameters reflect the continentality of the climate. Other two parameters, average temperature (Bio10) and precipitation (Bio18) of the warmest quarter, contributed 15.7% and 13.7% respectively. Despite the importance of coarse fragment content (Cfvo), it contributed only 10.3%. Precipitation of the driest month (Bio14) had the smallest contribution (5.1%).

**Figure 2:**
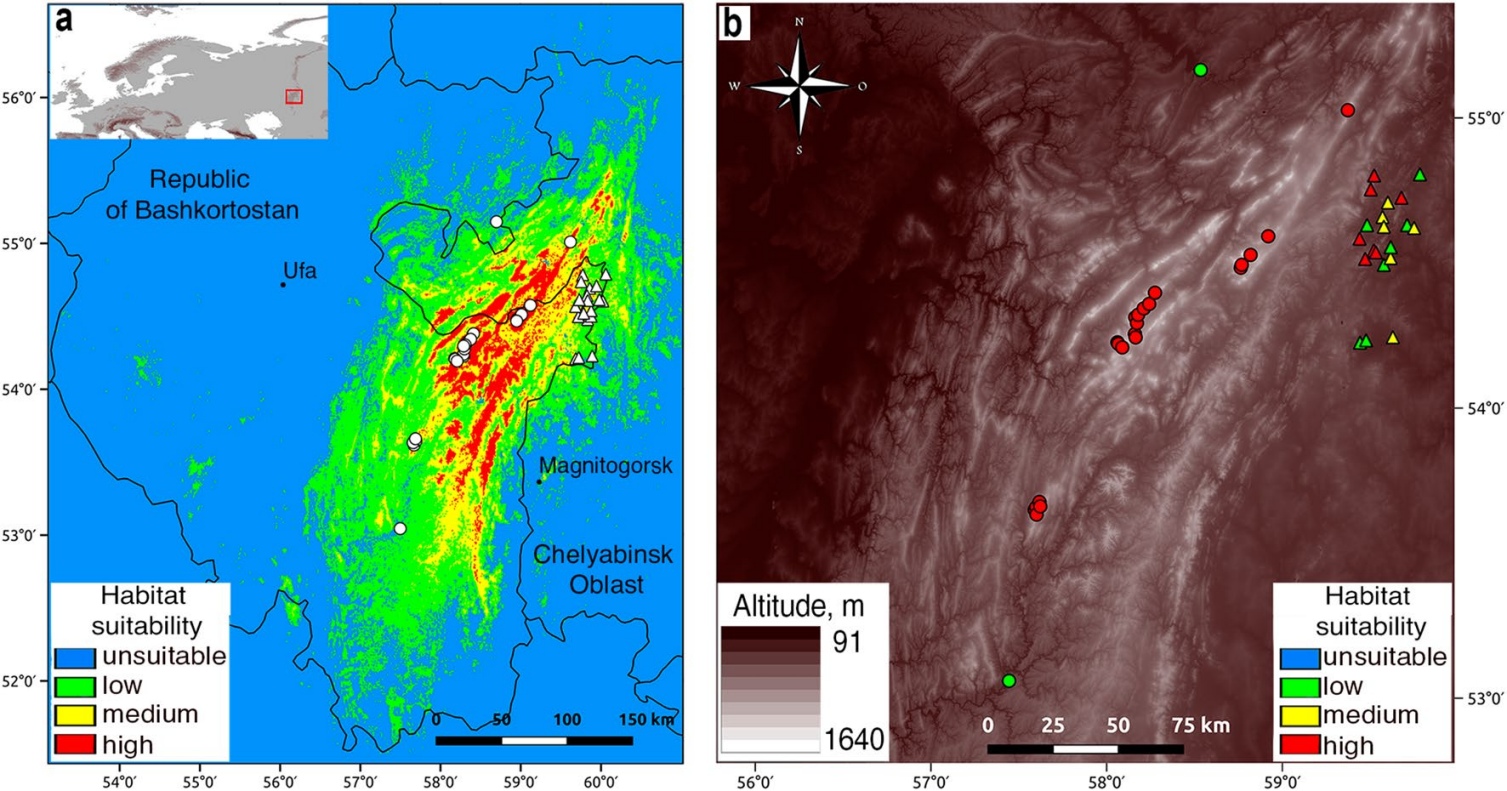
(**a**) The current potential range and locations of *Patrinia sibirica* in the Southern Urals; the location of the study area is shown in the upper left corner; (**b**) Elevation map and habitat suitability in the known locations of the species. Mountain forest locations are marked with circles, mountain forest-steppe locations are marked with triangles. The maps were created using QGIS (v.3.14; www.qgis.org) and GIMP (v.2.10.24; www.gimp.org).

Figure 2 shows the results of modeling of the habitat suitability for *P. sibirica* in the Southern Urals. The suitability was divided into 4 gradations: unsuitable habitats (0–0.34), low suitable habitats (0.35–0.60), medium suitable habitats (0.61–0.84) and high suitable habitats (0.85–1.00). The value 0.35 is the lowest limit of the habitat suitability for known locations of *P. sibirica*. Yet, the species was not registered in some areas of high suitability (Fig. 2a). An analysis of the habitat suitability in the known locations of *P. sibirica* has shown that 65% of the locations are high suitable, 14% are medium suitable, and 21% are low suitable. Most of medium and low suitable locations are situated in mountain forest-steppe areas except for two mountain forest locations. All locations are confined to rocky habitats. Coarse fragment content in the top layer of soil in mountain forest locations ranged from 15.4 to 22.5% and averaged 14.9%, and in mountain forest-steppe locations it ranged from 10.8 to 19.3%.

**Figure 3:**
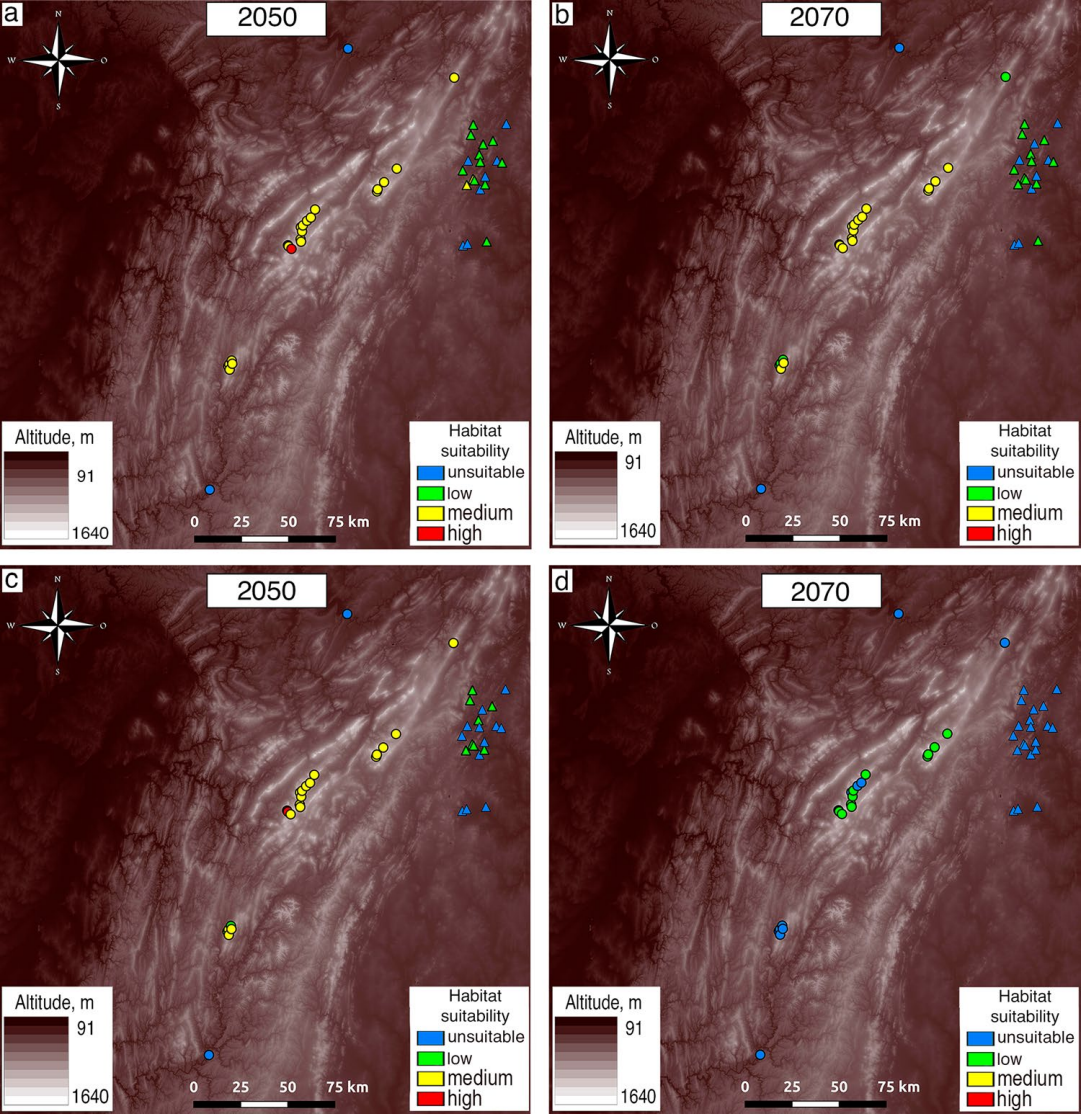
Predictions of the habitat suitability of the locations of *Patrinia sibirica* in the Southern Urals by 2050 and 2070 under the moderate (RCP4.5) (**a**, **b**) and the extreme (RCP8.5) (**c**, **d**) climate change scenarios. Mountain forest locations are marked with circles, mountain forest-steppe locations are marked with triangles. The maps were created using QGIS (v.3.14; www.qgis.org) and GIMP (v.2.10.24; www.gimp.org).

Figure 3 shows the predictions of the habitat suitability for *P. sibirica* in its known locations in the Southern Urals in 2050 and in 2070. Under the moderate climate change scenario (RCP4.5) the habitat suitability of mountain forest locations will decline below the threshold in two location (8.7% of the total number) by 2050 (Fig. 3a). Other mountain forest locations will be still suitable for *P. sibirica* by 2050 and 2070 (Fig. 3a, b). The habitat suitability of 35% of mountain forest-steppe locations will decline below the threshold by 2050; 40% of them will become unsuitable by 2070.

In mountain-forest locations under the extreme (RCP8.5), as well as the moderate (RCP4.5) climate change scenarios, habitat suitability is predicted to deteriorate to a critical level in 8.7% of locations by 2050 (Fig. 3c). At the same time, under the extreme climate change habitat suitability is predicted to decrease almost in half of the locations much more than under the moderate climate change by 2070 (Fig. 3d). A critical deterioration of the habitat suitability will occur in 60% of mountain forest-steppe locations by 2050, and all of the locations will become unsuitable by 2070 (Fig. 3d).

**Figure 4:**
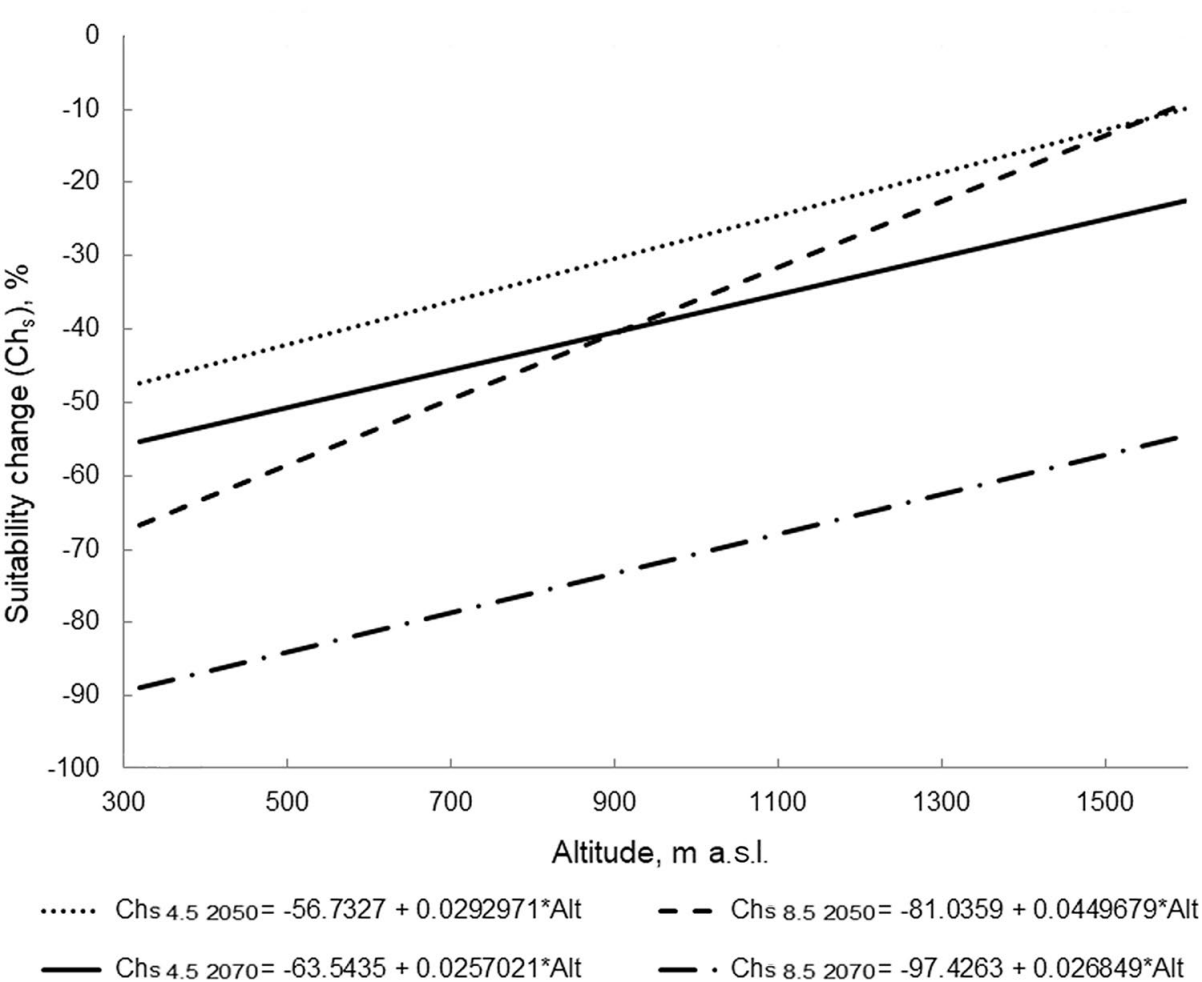
Results of linear regression analysis of the dependence of changes in habitat suitability (Ch_s_) in the Southern Urals locations of *Patrinia sibirica* on their altitude (Alt) under different climate change scenarios. Ch_s_ 4.5 and Ch_s_ 8.5 – habitat suitability changes under the RCP4.5 and RCP8.5 climate change scenarios.

The mountain forests locations are more resilient to climate changes than mountain forest-steppe locations. The former are on average larger in size and situated at different elevations. The correlations of the habitat suitability change (Ch_s_) with the area of locations were relatively low, ranging from 0.43 (RCP4.5, 2050) to 0.54 (RCP8.5, 2070). The correlation between habitat suitability changes and elevation under the RCP4.5 scenario by 2070 was 0.86; the correlations under other scenarios ranged between 0.94 and 0.97. Thus, linear regression models can be used to analyze the dependence of Ch_s_ in locations of *P. sibirica* on their altitude under climate change (Fig. 4).

Figure 4 shows the results of linear regression analysis of the dependence of changes in habitat suitability of the Southern Urals locations of *P. sibirica* on their elevation under various climate change scenarios. The slope of the line in the linear regression equation y = a + bx is reflected by the coefficient b. Thus, the line slope in the graph (Fig. 4) reflects the decline rate in the habitat suitability along the elevation gradient. Under the RCP4.5 scenario, the values of coefficient b of the lines of the habitat suitability change in 2050 and 2070 differ slightly. The slope of the line calculated for extreme climate change in 2070 is lower than in 2050, which may be partly related to the increasing role of the size of location, since 2070 is the period with the greatest correlation between their area and the changes in the habitat suitability. In 2050, the rates of change in habitat suitability along the elevation gradient under the extreme climate change scenario are higher than under the moderate climate change scenario. Meanwhile in 2070, the difference in rates of change of habitat suitability is decreasing.

The vegetation in most mountain forest-steppe locations of *P. sibirica* is petrophytic steppes adjoining or located close to areas of forest or shrub vegetation. These xerophyte habitats remain unchanged due to the soil hydrological characters which are unsuitable for forest vegetation. Table 1 shows that both temperature and precipitation will increase in these locations under climate change, most significantly in the driest quarter of a year (January-March). According to the forecast, the average precipitation of the driest quarter and the average annual precipitation by 2070 in the area of distribution of mountain-steppe locations will be approximately equal to the amount of precipitation in similar periods at present in the nearby forest areas, which are dominated by *Pinus sylvestris* L. and *Betula verrucosa* Ehrh.

**Table 1:** Average temperature and precipitation in the Southern Urals mountain forest-steppe locations of *Patrinia sibirica* at present and under different climate change scenarios and the current climatic parameters in closely located forests.

Scenarios	Average annual temperature, °C	Average precipitation, mm	
		Annual precipitation	Precipitation of driest quarter
Current	1.8	478.9	56.7
RCP4.5 (2050s)	4.3	519.3	63.0
RCP4.5 (2070s)	4.8	527.0	65.9
RCP8.5 (2050s)	4.9	487.5	61.1
RCP8.5 (2070s)	6.5	532.6	68.4
**Closely located forest areas**			
Current	1.7	544.8	63.7

## Discussion

Figure 2a shows that *P. sibirica* is absent in some areas identified as high suitable habitats according to the model of the current potential range. *P. sibirica* is not registered in the southern part of the eastern macroslope of the Southern Urals (the southeast part of the territory with high suitable habitats to the west from the city of Magnitogorsk). This is probably due to the fact that there was an expansion of forests during a more humid and warmer climate in the Holocene (about 5–6 thousand years ago). Therefore, a significant proportion of rocky open areas currently suitable for this species could have been occupied by other vegetation (primarily forest vegetation) in the past^15^. The model showed some relatively small areas which are low suitable for *P. sibirica* in the Bashkir Cis-Urals on the Belebeevskaya Upland in steppe and forest-steppe areas at altitudes higher than 400 m a.s.l. (southwest part of the map in Fig. 2b). *P. sibirica* does not grow in these areas probably due to the fact that most of the rock outcrops are fragile sandstones of the Permian period, on which petrophyte plants are almost completely absent because of soil instability.

The deterioration of habitat suitability in some locations of *P. sibirica* when the climate warms below the critical level (Fig. 3c, d) may be related to the competition from other plant species, inter alia, through the expansion of plant communities surrounding the habitats of the studied species. As noted above, elevation has a major influence on the persistence of the habitat suitability in the locations of *P. sibirica*. However, the role of the environmental factors changes along the elevation gradient. Winter and spring temperatures are limiting factors for *P. sibirica* in the mountain forest locations. At present, climate warming induces the expansion of forest vegetation including an upward shift of the upper boundaries of forests^16–18^. This may lead to a gradual decrease in size or even loss of some locations of *P. sibirica* in the subalpine altitudinal belts in the Southern Urals. The limiting factor for *P. sibirica* in its lowest locations in mountain forest-steppe areas is not temperature but aridity of climate in addition to grazing. Increased precipitation caused by climate change in mountain forest-steppe areas will lead to the invasion of forest (*Pinus sylvestris*, *Betula verrucosa*, *Larix sibirica* Ledeb.) and shrub vegetation (*Caragana frutex* (L.) K.Koch, *Cerasus fruticosa* Pall., *Spiraea crenata* L.) into the habitats of *P. sibirica*. That explains the predicted decline in the habitat suitability below critical levels in mountain forest-steppe areas under extreme climate change. This decline will not necessarily lead to a rapid pace of extinction of the species in these locations, as the expansion of forests may lag behind climate change^19^. Moreover, overgrowing of mountain forest-steppe locations of *P. sibirica* with forest and shrub vegetation may be slowed down if grazing is intensive.

The results allow prediction of the persistence of growth conditions for *P. sibirica* in the Southern Urals locations and explain reasons for the difference in their stability. Due to the climate scenario uncertainty^20^, the results should be considered as the most likely probabilities which require validation with the use of different methods^21^. The persistence of subalpine habitats of *P. sibirica* can be predicted based on the rate of change of the upper boundaries of the forest under climate change using retrospective Remote Sensing data sets. The persistence of mountain forest-steppe habitats is much more difficult to predict because the major factor in the formation of steppe vegetation is precipitation which has temporal (interannual) and random (cyclonic occurrences with anomalous precipitation) variability. However, the monitoring of the habitat persistence of relict species of the Pleistocene floristic complex and predictions of their changes are important, as their massive decline will mean that climate changes exceed ranges of their fluctuations in the Holocene. It is worth noting that to preserve the intraspecific diversity of *P. sibirica* in the Southern Urals in the case of an extreme climate change, reintroduction of plants from the most valuable mountain forest-steppe populations to more suitable habitats for the species is recommended. In this case, an analysis of the genetic diversity of vulnerable populations will be necessary to select material for the reintroduction.

## Materials and methods

### Data sources and variables selection

According to the herbarium records (UFA), Global Biodiversity Information Facility (GBIF)^22^ and literature sources^8,23^ there are 43 locations of *P. sibirica* with accurate georeferences known in the Southern Urals within the Republic of Bashkortostan and adjacent regions. Herbarium specimens were collected in the Southern Urals in 1925–2008. All specimens were identified by botanist and flora specialist A.A. Muldashev. The voucher specimens were deposited at the Herbarium of the Institute of Biology, Ufa Scientific Centre, Russian Academy of Sciences (UFA) (voucher numbers are: 1, 84, 106, 153, 159, 173, 182, 262, 270, 313, 331, 336, 338, 350, 359, 369, 370, 398, 413, 448, 466, 518, 520, 523, 564, 699, 723, 860, 901). The polygons of the locations and their centroids calculated in QGIS (v.3.14) on the basis of field research data have been used as the source material. Primary boundaries of the location polygons were established on the basis of georeferenced field data and then were corrected using high resolution space images. The final polygons cover the areas which are relatively homogeneous in relief and vegetation, where *P. sibirica* is found with varying population density. According to Karimova et al*.*^24^, the population density of this species varies from 1.2 to 6.5 plants/m^2^. However, according to our field studies, a wider range of the population density is possible.

In order to forecast the climate change impact on *P. sibirica,* its known locations were divided into two groups: 23 mountain forest locations and 20 mountain forest-steppe locations (Fig. 1). Twenty one mountain forest locations are situated in rocky remains or terraced ledges in the upper forest belt surpassing 950 m above sea level (m a.s.l.) (Mount Bolshoi Shatak) and in the subalpine and alpine altitudinal belts at the altitudes of up to 1600 m a.s.l. in the central elevated part of the Southern Urals. Other two locations are situated on the rocky shores of the Ay and Belaya rivers at the altitudes of 320 and 349 m a.s.l. respectively. Fifteen mountain forest locations are less than 1 hectare in size; other 8 locations occupy more than 4 hectares (the larger locations surpass 1250 m a.s.l.).

Mountain forest-steppe locations are found in petrophyte steppes at rock outcrops, on tops of small ridges and hills between 340 to 700 m a.s.l. Only two locations are situated on the higher rocky peaks of the Uchalinsky Irendyk Range at 760 m a.s.l. and 844 m a.s.l. Mountain forest-steppe locations are usually situated on narrow ridges of hills, often bordering on true steppes and shrub-steppes, as well as small areas of steppe forests. Almost all of them are less than 1 hectare in size, except for two locations: one is on the Uchalinsky Irendyk Range (1.28 hectare) and another one is on a rocky peak of a small ridge above the Shartymka River (3.46 hectare).

To extrapolate the models (for example, to forecasted climatic conditions), the samples used in modeling must be taken from the full range of environmental conditions, and their possible combinations must be sufficient to characterize the ecological niche of the species^25^. This can be achieved by combining samples from geographically and ecologically different regions into one sample^26^. For that, in addition to 43 locations in the Southern Urals, 40 additional georeferenced locations of *P. sibirica* in Western and Eastern Siberia were used to construct a model of the current potential range of *P. sibirica*. These data were taken from GBIF and from literature sources^27,28^.

### Environmental predictor variables and climate models

The set of BIOCLIM with 30 arc sec resolution in the WGS-84 coordinate system (the average size of a raster cell in the study area is 560 m from east to west and 920 m from north to south) from the CHELSA database^29,30^ was used as climate variables (predictors) in modeling (Supplementary Table S1). The habitat suitability was assessed for two climate change scenarios, RCP4.5 and RCP8.5. They correspond to atmospheric greenhouse gas concentrations of 650 p.p.m and 1370 p.p.m accordingly, i.e. to the moderate and extreme climate change. The periods 2040–2060 and 2060–2080 were denoted as 2050 and 2070 respectively. The model selection to assess the impact of the future climate was based on the recommendations^31,32^ and their successful employment for the Caucasus^33^, resulting in the use of the ensemble of four general circulation models: CCSM4^34^, INMCM4^35^, NorESM1-M^36^ and MIROC-ESM^37^.

Besides climatic variables, the characteristics of the digital elevation model GMTED2010 with the same 30 arc sec resolution as the climatic layers^38^ were used in modeling. Morphometric variables calculated by several neighboring pixels (slope, aspect, etc.) were not used because of the low resolution of the elevation layer and small size of locations. Thus, only an elevation layer (h_Mean_) was used in the model. In addition to the climatic variables listed above, to reflect the relationships of the species with the environment we also used the coarse fragment content (particles larger than 2 mm) in the top layer of soil (0–5 cm) from SoilGrids 2.0^39^. The original layer in the Goode's homolosine projection with 250 m resolution was reprojected in SAGA (v.7.7.0) to the WGS-84 coordinate system and resampled to the 30 arc sec resolution before modeling. The coarse fragment content in the soil layer most accurately characterizes the edaphic habitat conditions of this stenotopic species.

### Species distribution modeling

MaxEnt (v.3.4.1)^40^ was used for modeling of the habitat suitability in the locations of *P. sibirica* (which can be a value between 0 and 1) and its changes under climate warming^41^. We generated a Pearson’s correlation matrix of environmental predictors^42^. In the case of the correlation coefficient greater than or equal to 0.8, one of the variables was excluded to prevent multicollinearity and model overfitting^42,43^ (Supplementary Table S2). The AUC indicator was used for the statistical evaluation of the model^44,45^. MaxEnt standard tests (jackknife, permutation importance and percent contribution)^41^ were used to evaluate the reliability of contribution of the predictors to the model. The minimum value of the suitability in the known Southern Urals locations of the species at present was used as a threshold value of the habitat suitability.

Fivefold cross-validation was used to validate the predictive ability of the model, and the final model was the mean of the 5 folds. After MaxEnt calculations, the average resulting layer was calculated for each scenario and for every future time period using an ensemble of models. Average values of the habitat suitability in the Southern Urals locations were calculated in QGIS using the resulting raster layers. The habitat suitability changes (Ch_s_) in a climate change scenario in the locations were calculated as follows:

$${\text{Ch}}_{{\text{s}}} = \left( {{\text{S}}_{{\text{f}}} {-}{\text{S}}_{{\text{c}}} } \right)/{\text{S}}_{{\text{c}}} ,$$

where S_c_ is the average habitat suitability in the location at the current time, S_f_ is the average habitat suitability in the location in the review period of the corresponding climate change scenario.

## Supplementary Information

Supplementary Information.
