# Supplementary material for: Prediction of habitat suitability for Patrinia sibirica Juss. in the Southern Urals
Source: Sci Rep. 2021 Oct 4;11:19606. doi: 10.1038/s41598-021-99018-0 (PMC8490377; doi:10.1038/s41598-021-99018-0)
Supplement: Supplementary file 1 — Supplementary Information. [file 41598_2021_99018_MOESM1_ESM.pdf]

Table S1. The estimates of relative contributions of the environmental variables to the MaxEnt model.

Code/Unit	Variable	Percent contribution	Permutation importance
**bio3 (%)**	**Isothermality**	**21.5**	**33.6**
h_{Mean} (m)	$Altitude_{Mean}$	19.3	4.6
**bio4 (°C)**	**Temperature Seasonality**	**18.4**	**22.6**
**bio18 (mm)**	**Precipitation of Warmest Quarter**	**12.6**	**0.6**
bio11 (°C)	Mean Temperature of Coldest Quarter	6.9	0
bio17 (mm)	Precipitation of Driest Quarter	5.8	1.2
**bio10 (°C)**	**Mean Temperature of Warmest Quarter**	**3.8**	**24.7**
bio7 (°C)	Temperature Annual Range	3.3	8.9
bio14 (mm)	Precipitation of Driest Month	3.1	0
bio9 (°C)	Mean Temperature of Driest Quarter	2.1	0.6
bio15 (CV)	Precipitation Seasonality	1.3	0.9
bio2 (°C)	Mean Diurnal Range	0.7	1.1
bio12 (mm)	Annual Precipitation	0.6	0.1
bio5 (°C)	Max Temperature of Warmest Month	0.2	0.7
bio19 (mm)	Precipitation of Coldest Quarter	0.2	0
**Cfvo (%)**	**Coarse fragments**	**0.1**	**0.2**
bio16 (mm)	Precipitation of Wettest Quarter	0.1	0.2
bio8 (°C)	Mean Temperature of Wettest Quarter	0.1	0.1
bio1 (°C)	Annual Mean Temperature	0	0
bio13 (mm)	Precipitation of Wettest Month	0	0

bio6 (°C)	Min Temperature of Coldest Month	0	0

Note: Ecological predictors left after removal of predictors with high pair correlation are marked in bold.

Table S2. Correlation analysis of the environmental variables.

Variables	bio1	bio2	bio3	bio4	bio5	bio6	bio7	bio8	bio9	bio10	bio11	bio12	bio13	bio14	bio15	bio16	bio17	bio18	bio19	Mean	Min-Max
bio1																					
bio2	-0,48																				
bio3	-0,39	0,80																			
bio4	0,08	0,09	-0,51																		
bio5	0,97	-0,38	-0,44	0,32																	
bio6	0,91	-0,66	-0,33	-0,28	0,78																
bio7	-0,27	0,59	-0,01	0,84	-0,03	-0,65															
bio8	0,85	-0,48	-0,53	0,28	0,87	0,71	-0,07														
bio9	0,89	-0,59	-0,38	-0,14	0,81	0,91	-0,47	0,78													
bio10	0,97	-0,44	-0,50	0,32	1,00	0,79	-0,05	0,88	0,82												
bio11	0,94	-0,53	-0,23	-0,26	0,83	0,98	-0,57	0,73	0,91	0,83											
bio12	0,04	-0,47	-0,30	-0,18	-0,03	0,21	-0,37	-0,13	0,03	-0,01	0,10										
bio13	-0,18	-0,43	-0,39	-0,02	-0,22	-0,06	-0,18	-0,19	-0,20	-0,18	-0,17	0,86									
bio14	0,34	-0,57	-0,31	-0,24	0,24	0,51	-0,52	0,08	0,35	0,25	0,41	0,86	0,53								
bio15	-0,60	0,60	0,31	0,26	-0,50	-0,74	0,58	-0,34	-0,61	-0,50	-0,67	-0,68	-0,31	-0,89							
bio16	-0,18	-0,42	-0,37	-0,03	-0,22	-0,06	-0,18	-0,19	-0,20	-0,18	-0,17	0,88	1,00	0,54	-0,33						
bio17	0,33	-0,57	-0,33	-0,22	0,24	0,50	-0,51	0,08	0,33	0,25	0,40	0,87	0,56	1,00	-0,89	0,58					
bio18	-0,17	-0,44	-0,38	-0,06	-0,22	-0,03	-0,22	-0,17	-0,18	-0,18	-0,15	0,88	0,99	0,54	-0,34	1,00	0,57				
bio19	0,22	-0,48	-0,31	-0,13	0,16	0,35	-0,37	-0,02	0,20	0,17	0,26	0,92	0,64	0,96	-0,82	0,67	0,97	0,65			

h_{Mean}	-0,97	0,62	0,59	-0,19	-0,95	-0,88	0,24	-0,86	-0,87	-0,97	-0,88	-0,15	0,03	-0,40	0,63	0,03	-0,40	0,02	-0,31		
Cfvo	-0,57	-0,13	-0,16	-0,02	-0,57	-0,42	-0,03	-0,38	-0,44	-0,54	-0,52	0,07	0,29	-0,10	0,32	0,28	-0,09	0,29	-0,07	0,46	0,41

Note: If two variables had r >±0.8, only one of them was selected in the same model;
